# Type A Acute Aortic Dissection Presenting With Arch and Abdominal Aortic Aneurysms

**DOI:** 10.7759/cureus.44629

**Published:** 2023-09-04

**Authors:** Hideki Sasaki, Yoshiaki Sone, Shinji Kamiya, Yukihide Numata, Syunta Hayakawa

**Affiliations:** 1 Cardiovascular Surgery, Nagoya City University East Medical Center, Nagoya, JPN

**Keywords:** emergency department, computed tomography, percutaneous coronary intervention, abdominal aortic aneurysm, thoracic aortic aneurysm, acute aortic dissection

## Abstract

Herein, we present the case of an 89-year-old female who presented with acute aortic dissection involving the arch and abdominal aneurysms. Emergent total arch replacement with a frozen elephant trunk was the first-line approach taken to save the patient's life. Although prolonged mechanical ventilation necessitated a tracheostomy, subsequent endovascular aortic repair mitigated the risk of rupture in the abdominal aortic aneurysm. While managing acute aortic syndrome with multiple aneurysms poses a challenge for surgeons, a diagnosis based on computed tomography angiography and timely intervention alleviated the critical condition.

## Introduction

Type A acute aortic dissection (AAAD) is a life-threatening condition that requires prompt diagnosis and treatment. AAAD occurs in three to five out of every 100,000 individuals in the population per year, with a mortality rate of 1-2% per hour. In our current aging society, octogenarians are increasingly undergoing surgery for AAAD [1,2]. Furthermore, elderly patients often have more comorbidities and greater frailty. When a patient presents with AAAD along with multiple aortic aneurysms, meticulous attention and timely management become crucial. In this case, we present the situation of an 89-year-old woman who had AAAD along with aortic arch and abdominal aortic aneurysms.

## Case presentation

**Figure 1:**
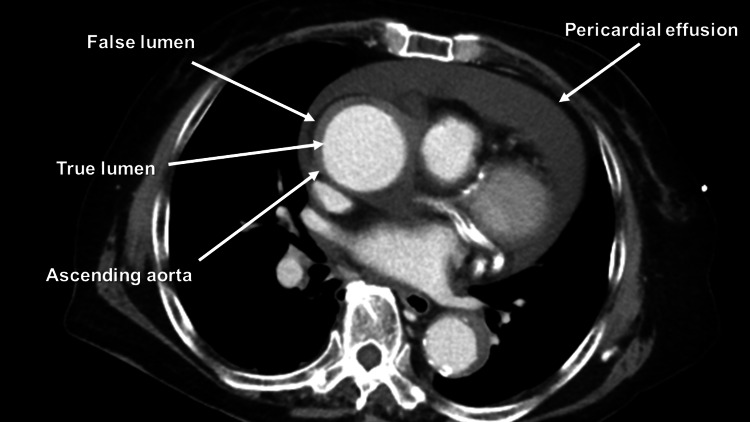
Preoperative contrast-enhanced computed tomography Preoperative contrast-enhanced computed tomography revealing type A acute aortic dissection and pericardial effusion

**Figure 2:**
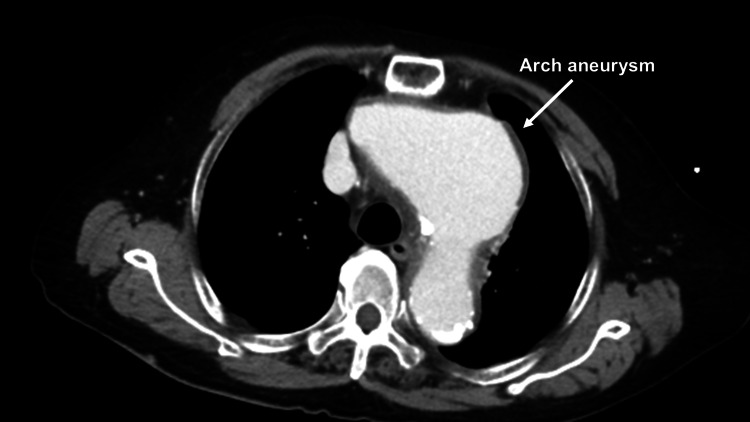
Preoperative contrast-enhanced computed tomography Preoperative contrast-enhanced computed tomography revealing an arch aneurysm

**Figure 3:**
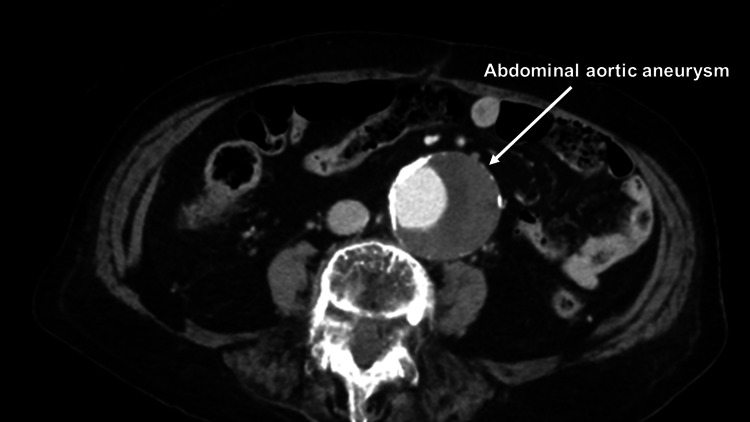
Preoperative contrast-enhanced computed tomography Preoperative contrast-enhanced computed tomography revealing abdominal aortic aneurysm

An 89-year-old female presented to the emergency department with complaints of back pain and shortness of breath. The patient had a medical history of undergoing percutaneous coronary intervention (PCI) for the right coronary artery and left circumflex artery two months ago, as well as having hypertension and chronic kidney disease (CKD) with a calculated estimated glomerular filtration rate (eGFR) of 35. She was prescribed prasugrel hydrochloride 3.75 mg/day and aspirin 100 mg/day. Additionally, arch and abdominal aortic aneurysms had been previously detected. Although both the arch aneurysm and abdominal aortic aneurysm were large, indicating the need for surgery, the patient and her family hesitated due to the patient’s age. The patient’s blood pressure was 64/49 mmHg with a heart rate of 70/min. Contrast-enhanced computed tomography (CECT) revealed pericardial effusion, type A acute aortic dissection (AAAD), a 62 mm-sized arch aneurysm, and a 55 mm-sized abdominal aortic aneurysm (AAA) (Figures 1-3).

**Figure 4:**
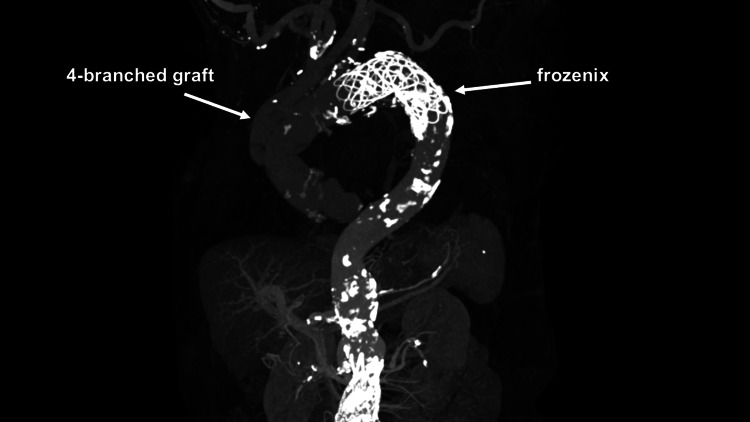
Postoperative computed tomography angiography Postoperative computed tomography angiography revealing excluded arch aneurysm

**Figure 5:**
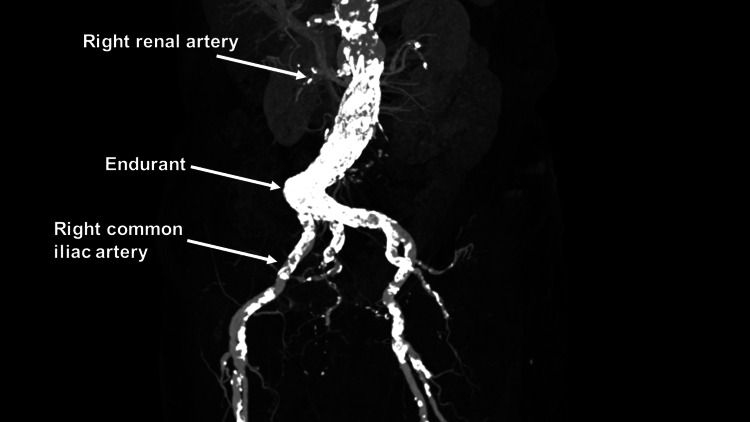
Postoperative computed tomography angiography Postoperative computed tomography angiography revealing excluded abdominal aortic aneurysm

Given the emergent situation, we decided to perform a total arch replacement (TAR) as a life-saving operation. The patient underwent the procedure under general anesthesia, with a median sternotomy performed. Cardiopulmonary bypass (CPB) was established using ascending aortic perfusion and right atrium drainage. After reducing the patient's body temperature to 23 degrees Celsius, circulatory arrest was induced, and cardioplegia was administered through the coronary sinus. Following the attainment of cardiac arrest, an intimal tear was identified at the lesser curvature of the aortic arch. The aorta was transected between the innominate artery (INA) and the left common carotid artery (LCCA). After preparing the distal anastomosis site, a Frozenix J graft (Japan Lifeline, Tokyo, Japan) measuring 29 mm in diameter and 120 mm in length was inserted into the aortic arch as a frozen elephant trunk (FET) to exclude the aortic arch aneurysm. A 28mm 4-branched graft was then anastomosed to the distal anastomosis site, with reconstructions of the left subclavian artery (LSCA), LCCA, and INA using separate branches of the graft. Following the anastomosis of the proximal graft to the ascending aorta, the aortic cross-clamp was released, and the heart resumed spontaneous sinus rhythm. The patient was successfully weaned from CPB and transferred to the intensive care unit (ICU) in stable condition. However, the postoperative course was complicated by respiratory failure. Despite multiple attempts to discontinue ventilator support during the first postoperative week, we encountered challenges due to hypercapnia. The patient had to be re-intubated three times after removing the endotracheal tube, as persistent hypercapnia remained a concern. Subsequently, a tracheostomy was performed on the eighteenth postoperative day. While the hypercapnia persisted, it gradually improved. After extensive discussions with the patient and her family, a decision was made to perform endovascular aortic repair (EVAR; EndurantTM IIs 32 mm, Medtronic, Minneapolis, Minnesota) for AAA and to conduct coil embolization of the third and fourth lumbar arteries on the seventy-sixth postoperative day. Following these procedures, the tracheostomy tube was removed on the seventy-seventh postoperative day. Postoperative CT revealed satisfactory results (Figures 4, Figure 5).

The patient was eventually transferred to a rehabilitation facility on the hundred and ninth postoperative day.

## Discussion

AAAD is a life-threatening condition that requires prompt diagnosis and treatment. In the current case, the patient was elderly and the case was complicated by an arch aneurysm and AAA. In this situation, the attending surgeon needs to initiate a conversation with the patient and her family and promptly decide on the operative procedure. There are three key points to discuss in this case. The first point pertains to the decision regarding the initial operative procedure in this emergent situation. The second point addresses the management of prolonged mechanical ventilation after surgery, and the third point involves the decision-making process for the AAA procedure. Regarding the first point, the decision for the initial operative procedure for AAAD presents complexities. The patient, an 89-year-old with hemodynamic collapse and a recent history of PCI while on dual antiplatelet therapy (DAPT), poses a challenge. Although there are reports on AAAD repair for octogenarians, the preference leans toward less invasive surgery given the urgency of the situation [3]. Considering the patient's age and fragile tissue, the primary concern for the surgeon revolves around the bleeding tendency associated with coagulopathy after CPB and DAPT. Despite these concerns, a decision was made to proceed with TAR using FET. The advantage of FET lies in the proximity of the distal anastomosis site to the TAR, making it easier for surgeons to identify bleeding points [4]. Another concern involves the choice of perfusion site during the establishment of CPB. Given the patient's AAA condition, femoral artery perfusion could potentially lead to retrograde embolism such as brain infarction. Although the right subclavian artery is a commonly used perfusion site in AAAD cases [5], its exposure requires additional time. Given the readily accessible ascending aorta, it was utilized in this case. The second challenge encountered was prolonged mechanical ventilation. Despite multiple attempts in the first postoperative week, we faced difficulty in weaning the patient off the ventilator due to hypercapnia. It was hypothesized that perioperative fluid retention, compounded by CKD, contributed to persistent hypercapnia. This necessitated an extended period for improvement. Consequently, a tracheostomy was performed on the eighteenth postoperative day. Although an additional 59 days were required, a gradual improvement in hypercapnia was observed, leading to the eventual removal of the tracheostomy tube. The third point of discussion involves the procedure for the AAA. With the patient's condition gradually improving over time, we confirmed both the patient's and the family's wishes and decided to proceed with the surgery. Following comprehensive discussions involving interventional radiologists and cardiovascular surgeons, we opted for EVAR for AAA. Taking into account factors such as the patient's age, the post-AAAD repair status, and the need for prolonged mechanical ventilation, EVAR was deemed a suitable choice. In the current era, endovascular surgery is prevailing. Thoracic endovascular aortic repair (TEVAR) combined with EVAR is reported to be useful in treating a patient with simultaneous thoracic and abdominal aortic aneurysms [6-8]. Although simultaneous repair for arch and abdominal aortic aneurysms is not feasible in the current case, endovascular surgery can give surgeons more options for this kind of patient with multiple aortic aneurysms.

## Conclusions

When an elderly patient presents with aortic and abdominal aneurysms complicated by AAAD, the physician needs to prioritize performing a life-saving procedure for AAAD.

Total arch replacement with the frozen elephant technique is the first-line procedure, which can reduce the risk of bleeding at the distal anastomosis. Tracheostomy is a valid option for patients experiencing persistent hypercapnia and prolonged mechanical ventilation. EVAR for AAA is a viable choice considering the patient’s post-AAAD repair status.
